# *TNF-α* –308 G>A and –238 G>A polymorphisms are not major risk factors in Caucasian patients with exfoliation glaucoma

**Published:** 2009-03-09

**Authors:** Georg Mossböck, Wilfried Renner, Yosuf El-Shabrawi, Christoph Faschinger, Otto Schmut, Andreas Wedrich, Christina Zimmermann, Martin Weger

**Affiliations:** 1 Department of Ophthalmology, Medical University Graz, Austria; 2 Clinical Institute of Medical and Chemical Laboratory Diagnostics, Medical University Graz, Austria

## Abstract

**Purpose:**

TNF-α has been suggested to participate in the pathogenesis of exfoliation glaucoma (XFG). The purpose of the present study was to investigate a hypothesized association between two common functional polymorphisms in the promoter region of the *TNF-α* gene (*TNF-α *–308 G>A, rs1800629, and *TNF-α *–238 G>A, rs361525) and the presence of XFG in a Caucasian population.

**Methods:**

The present case-control study comprised 408 participants (204 patients with XFG and 204 control subjects). Control subjects were matched for age and sex. Genotypes of the *TNF-α* –308 G>A and *TNF-α* –238 G>A polymorphisms were determined by polymerase chain reaction (restriction fragment length polymorphism).

**Results:**

No significant differences regarding genotype distribution or allelic frequencies were found between patients and control subjects (p>0.025). The presence of the *TNF-α* –308 G-allele was associated with an insignificant odds ratio of 0.98 (95% confidence interval [CI]: 0.66–1.46; p=0.99) while the presence of the *TNF-α* –238 G-allele was associated with an insignificant odds ratio of 0.64 (95% CI: 0.33–1.23; p=0.25).

**Conclusions:**

Our data suggest that both the *TNF-α* –308 G>A and the *TNF-α* –238 G>A polymorphisms are unlikely to be major risk factors for XFG in an European population of Caucasian descent.

## Introduction

Exfoliation syndrome (XFS; OMIM 177650) is recognized as a general elastic fibrillopathy, leading to accumulation of characteristic abnormal fibrillar material in different intra- and extraocular tissues, e.g., trabecular meshwork, iris stroma, conjunctiva, kidney, lung, and heart [1,2]. Glaucomatous optic neuropathy due to XFS (exfoliation glaucoma, XFG) is caused by deposition of exfoliation material and liberated iris pigment in the trabecular meshwork leading to elevation of intraocular pressure [3]. Results from family-based studies and from a study investigating the loss of heterozygosity in specimens of the anterior segment in individuals with XFS indicate a high grade of heritability of XFS [4-8]. Recently, two common non-synonymous single nucleotide polymorphisms (SNPs; rs1048661 and rs3825942) in the lysyl oxidase-like protein 1 gene (*LOXL1*; OMIM 153456) that confer increased risk for the development of XFS and XFG have been identified [9]. However, as the prevalences of these polymorphisms are also high in individuals without XFS, other, as yet unkown genetic or enviromental factors may be involved [10-16].

The degradation of the extracellular matrix (ECM) depends on the activity of a group of highly regulated endopeptidases named matrix metalloproteinases (MMPs), which are grouped in four families consisting of 23 members in total, each with a specific spectrum of ligands [17]. Uncontrolled activation of MMPs is counterbalanced by specific tissue inhibitors of metalloproteinases (TIMPs), and a delicate balance of MMPs and TIMPs is required for physiologic ECM turnover. Interestingly, increased levels of TIMP-1 and TIMP-2 and decreased levels of MMP-2 have been detected in the aqueous humor of patient with XFS [18,19]. An impaired balance between MMPs and TIMPs may thus contribute to the development of XFS.

Tumor necrosis factor-alpha (TNF-α, OMIM 191160) is a ubiquitous cytokine involved in various physiologic as well as pathologic processes like inflammation, immunoregulation, proliferation, and apoptosis [20]. Notably, several in vitro studies provided evidence that TNF-α is also capable of inducing both increased synthesis and activity of MMPs [21-24].

Two common functional polymorphisms in the promoter region of *TNF-α* have been identified. The first is characterized by a G to A substitution at position –308 (*TNF-α* –308 G>A, rs1800629) while the second is characterized by a G to A substitution at position −238 (*TNF-α* –238 G>A, rs361525). In vitro studies of these polymorphisms have been shown to increase TNF-α production after lipopolysaccharide stimulation [25-27]. Other studies, however, were unable to confirm this effect on TNF-α synthesis [27-29].

Only recently, Tekeli and coworkers [30] found an increased prevalence of the *TNF-α* −308 GG genotype among 110 Turkish patients with XFG. This finding, however, has not yet been confirmed in other populations, which is mandatory to draw firm conclusions on the role of *TNF-α* polymorphisms. Our study was set to investigate a hypothesized association between the *TNF-α* –308 G>A and *TNF-α* –238 G>A polymorphisms and the presence of XFG.

## Methods

Two hundred and four unrelated patients with XFG and 204 control subjects were enrolled in the present retrospective case-control study. All study participants were Caucasians from the same geographical area in the southern part of Austria. Participants were seen at the Department of Ophthalmology, Medical University Graz (Graz, Austria) between May 2003 and September 2008 and gave informed written consent before enrollment. The study was conducted in accordance with the National Gene Technology Act of Austria and the guidelines of the local ethics committee.

Patients with XFG underwent slit lamp biomicroscopy in mydriasis, testing for best corrected visual acuity, Goldmann applanation tonometry, gonioscopy, pachymetry, and standard automated perimetry (Octopus 101, program G2; Interzeag, Schlieren, Switzerland) or in cases of profoundly decreased visual acuity, Goldmann perimetry. Control subjects underwent slit lamp biomicroscopy in mydriasis, Goldmann applanation tonometry, and testing for best corrected visual acuity. Optic discs in all participants were assessed by glaucoma specialists (C.F., G.M.).

XFG was defined by the presence of typical exfoliation material on the anterior lens capsule, an intraocular pressure before initiation of a pressure lowering therapy of at least 22 mmHg, an open anterior chamber angle, optic disc changes characteristic for glaucoma (notching, thinning of the neuroretinal rim, increased cup/disc ratio in relation to the optic disc size), visual field defects characteristic for glaucoma (inferior or superior arcuate scotoma, nasal step, paracentral scotoma), and absence of conditions leading to secondary glaucoma.

Control subjects showed biomicroscopically no evidence of exfoliation material on the anterior capsule of the lens and no morphological damage indicative for open-angle or angle closure glaucoma. Control subjects were admitted to our department for cataract surgery.

### Genotype determination

Venous blood was collected in 5 ml EDTA tubes. DNA was isolated using QIAamp DNA blood mini-kit (QIAGEN, Venlo, Netherlands) and stored at −20 °C. All polymerase chain reactions (PCRs) were run under conditions previously described [31]. Primer sequences for the gene polymorphism at –308 were forward 5′-GGG ACA CAC AAG CAT CAA GG-3′ and reverse 5′-GGG ACA CAC AAG CAT CAA GG-3′, for the polymorphism at −238 forward 5′-ATC TGG AGG AAG CGG TAG TG-3′ and reverse 5′-AGA AGA CCC CCC TCG GAA CC-3′. DNA samples were amplified in 25 µl aliquots containing 200 µM deoxynucleoside triphosphate, 10 µM of each primer, 1.5 mM MgCl_2_, 1 µl DNA sample, and 2 U Taq polymerase (Applied Biosystems, Foster City, CA). Annealing temperature was 62 °C. The PCR products were digested at 37 °C with NcoI to detect the SNP in the −308 gene allele and MspI to detect the polymorphism of the −238 nucleotide. The PCR product was then subjected to 3% agarose-gel electrophoresis. “No target” controls were included in each PCR batch to ensure that reagents had not been contaminated.

### Statistical analysis

Descriptive statistics were used to calculate frequencies and percentages of discrete variables. Continuous data are given as mean±standard deviation (SD). Means were compared using the Mann–Whitney test. Proportions of groups were compared by the χ^2^ test. The odds ratio (OR) and 95% confidence interval (95% CI) were calculated by logistic regression. According to the Bonferroni correction, the criterion for statistical significance was set at p≤0.025. Hardy–Weinberg equilibrium has been calculated using HW Diagnostics-Version 1.beta (Fox Chase Cancer Center, Philadelphia, PA). Statistical analysis was done using the SPSS statistical package (version 14.0; SPSS, Chicago, IL).

## Results

The present study comprised of 204 patients with XFG (118 female and 86 male) and 204 control subjects, all matched for age and gender. The mean age of patients with XFG was 76.1±7.0 years (range: 60.6–91.7 years), and the mean age for control subjects was 75.3±6.9 years (range: 60.3–93.1 years).

The observed genotype distributions did not deviate from those predicted by the Hardy–Weinberg equilibrium, and for control subjects, they were similar to those reported for Caucasian populations [32]. Table 1 shows the genotype and allele frequencies of *TNF-α* –308 G>A and *TNF-α* −238 G>A polymorphisms in patients with XFG and control subjects. No significant differences in either genotype distribution or allelic frequencies of the *TNF-α* –308 G>A and the *TNF-α* −238 G>A polymorphisms were found between patients with XFG and control subjects. The presence of the *TNF-α* –308 G-allele was associated with an insignificant odds ratio of 0.98 (95% CI: 0.66–1.46; p=0.99) for XFG. An insignificant odds ratio of 0.64 (95% CI: 0.33–1.23; p=0.25) for XFG was also found among carriers of the *TNF-α* –238 G-allele. The present study had a statistical power of 0.80 to detect an odds ratio ≥2.09 for the *TNF-α* –308 G/G genotype and an odds ratio ≥4.19 for the *TNF-α* −238 G/G genotype in patients with XFG.

**Table 1:** Distribution of genotypes and allelic frequencies.

*TNF-α* single nucleotide polymorphism	Patients with XFG (n=204)	Control subjects (n=204)	Significance p-value
*TNF-α* –308GG	152 (74.5)	151 (74.0)	0.91
–308GA	48 (23.5)	51 (25.0)	0.73
–308AA	4 (2.0)	2 (1.0)	0.41
*TNF-α* –308 G allele frequency	0.863	0.865	0.92
*TNF-α* –238GG	182 (89.2)	189 (92.6)	0.23
–238GA	21 (10.3)	15 (7.4)	0.30
–238AA	1 (0.5)	-	-
*TNF-α* –238 G allele frequency	0.944	0.963	0.18

Distribution of *TNF-α* –308 G>A and *TNF-α* –238 G>A genotypes and allelic frequencies in patients with exfoliation glaucoma and control subjects in an European population of Caucasian descent. Numbers for genotypes are n (%).

## Discussion

TNF-α has been shown to affect activity and synthesis of MMPs and TIMPs, which play a major role in the turnover of the ECM in the trabecular meshwork. Treatment of rat cardiac fibroblasts with TNF-α exhibited an increased activity of MMP-2, MMP-9, and MMP-13, while stimulation of human smooth muscle cells led to increased activity of MMP-1, MMP-3, and MMP-9 [21,24]. Furthermore, in an in vitro study using human endothelial cells, TNF-α induced increased levels of MMP-14, which in turn activated latent MMP-2 [22]. In mouse osteoblastic cells, mRNA levels of *MMP-3*, *MMP-9*, *TIMP-1*, and *TIMP-3* were upregulated by TNF-α [23].

Various studies provided evidence that MMPs and TIMPs may be involved in the pathogenesis of XFG. Schlötzer-Schrehardt and coworkers found a decreased ratio of MMP-2 to TIMP-2 in the aqueous humor of patients with XFG, which has been corroborated by Määttä and coworkers [18,19]. Ho and coworkers [33] reported significantly increased levels of TIMP-1 in the aqueous humor of patients with XFS, while in an immunohistochemical study Rönkkö and coworkers [34] found decreased ratios of MMPs to TIMPs in trabecular meshwork specimens of patients with XFG. Remarkably, Tekeli and coworkers [30] investigating the common functional *TNF-α* –308 G>A polymorphism suggested that this polymorphism may exert protection against XFG. In their study that included 110 patients with XFG, 6.4% of the patients with XFG were found to have the heterozygote *TNF-α* –308 GA genotype compared to 16.4% of the control subjects. In the present study, no significant differences were found in the genotype distribution or allelic frequency of the *TNF-α* –308 G>A polymorphism as was the case for the *TNF-α* −238 G>A polymorphism. An insignificant odds ratio for XFG was found in both carriers of the *TNF-α* −308 G-allele and of the *TNF-α* −238 G-allele, suggesting that these polymorphisms themselves are not major risk factors for XFG. Possible explanations for these conflicting results may include small sample size as well as varying genotype distributions among different populations.

A limitation of the present study is the sample size, whereby minor genetic effects may be undetected. Furthermore, it can not be ruled out that other, as yet unknown functional polymorphisms in the TNF-α gene influence susceptibility to XFG.

The *TNF-α* –308 G>A polymorphism has also been recently implicated in the pathogenesis of primary open-angle glaucoma. An increased prevalence of the *TNF-α* −308 A-allele was reported in patients with primary open-angle glaucoma in a Chinese study, albeit this result could not be replicated in subsequent studies [35-37].

In conclusion, based on the results of the present study, neither the *TNF-α* –308 G>A nor the *TNF-α* −238 G>A polymorphism is a major risk factor among Caucasian patients with XFG.
